# Incidence and predictors of difficult videolaryngoscopy using a hyperangulated device in elective surgical patients: a prospective cohort study in China

**DOI:** 10.1186/s12871-025-03496-y

**Published:** 2025-11-25

**Authors:** Hailong Bing, Guanyu Yang, Hongwei Wang, Yili Zhao, Liumei Li, Zeping Li, Jianjun Yang, Qinjun Chu

**Affiliations:** 1 Department of Anesthesiology and Perioperative Medicine, Zhengzhou Central Hospital Affiliated to Zhengzhou University, Zhengzhou, Henan China; 2 Department of Anesthesiology and Perioperative Medicine, The First Affiliated Hospital of Zhengzhou University, Zhengzhou, Henan China; 3 Office of Work Style Construction, Zhengzhou Central Hospital Affiliated to Zhengzhou University, Zhengzhou, Henan China

**Keywords:** Airway management, Laryngoscopy, Mallampati score, Intubation, Intratracheal, Risk factors

## Abstract

**Background:**

While the utilization of video laryngoscope has become increasingly prevalent, the incidence of difficult videolaryngoscopy and its associated factors remain largely undefined. Therefore, this study was undertaken to determine the incidence of difficult videolaryngoscopy using a hyperangulated device, and to identify associated factors, specifically within a cohort of elective surgical patients in China presenting with normal airway anatomy.

**Methods:**

This study was a prospective cohort investigation that included 4,902 adult patients who underwent single-lumen tracheal intubation using a video laryngoscope. Data encompassing participants’ demographic characteristics, preoperative airway assessments, and intubation procedure details were systematically collected. Patients were categorized into two groups based on the Cormack-Lehane classification, with difficult videolaryngoscopy defined as a Cormack-Lehane grade of ≥ 3. The incidence of difficult videolaryngoscopy was documented. Subsequently, logistic regression analysis was performed to investigate factors associated with its occurrence.

**Results:**

The findings indicated that the incidence of difficult videolaryngoscopy was 2.1%. Multivariate logistic regression analysis demonstrated a statistically significant effect of interincisor distance on the incidence of difficult videolaryngoscopy (OR = 0.669, 95% CI = 0.490–0.913, *P* = 0.011). Additionally, a statistically significant association was found between Mallampati grade III-IV and difficult videolaryngoscopy (Mallampati grade III: OR = 3.200, 95% CI = 1.813–5.650, *P* < 0.001; Mallampati grade IV: OR = 2.899, 95% CI = 1.567–5.361, *P* = 0.001).

**Conclusions:**

The incidence of difficult videolaryngoscopy was 2.1% in this cohort when using a hyperangulated videolaryngoscope. The interincisor distance and Mallampati grades (III and IV) are independently predictors for difficult videolaryngoscopy with this blade type.

**Trail registration:**

www.chictr.org.cn/. Identifier ChiCTR220058009, 26 March 2022.

## Background

Difficult airway management remains a critical challenge in anesthesiology and is a leading cause of anesthesia-related morbidity and mortality [1]. Analyses of closed malpractice claims have consistently identified difficult tracheal intubation as a major contributor to adverse outcomes [2]. Furthermore, large national audits have revealed that complications during airway management, particularly in cases of unanticipated difficulty, can have devastating consequences [3]. Video laryngoscopes (VL) are emerging tools in airway management, with multiple studies demonstrating that they can enhance intubation success rates compared to traditional direct laryngoscopes [4–7]. Consequently, there is an increasing advocacy for the use of VL as a replacement for direct ones.

Prior research has predominantly concentrated on the incidence and associated factors of difficult laryngoscopy [8–11]. With the adoption of VL, focus has shifted to understanding challenges with this new technology. Contemporary guidelines have begun to identify factors associated with difficult tracheal intubation when using a VL [12]. However, robust, large-scale data specifically addressing the incidence and predictors of difficult glottic exposure with VL itself (i.e., difficult videolaryngoscopy, defined as a Cormack-Lehane grade of ≥ 3) remain scarce. Therefore, this study was undertaken to systematically evaluate the incidence of difficult videolaryngoscopy and to identify its associated factors in a broad population of patients with normal airway anatomy.

## Methods

This study was a prospective cohort study conducted across two centers: Zhengzhou Central Hospital Affiliated to Zhengzhou University and the First Affiliated Hospital of Zhengzhou University. The Ethics Committee (Chair: Yueping Wu) of the Zhengzhou Central Hospital approved this study on 18 March 2022 (reference number: 202235). The Ethics Committee of the First Affiliated Hospital of Zhengzhou University approved an ethical waiver due to the observational nature of the study. The research was registered with the Chinese Clinical Trial Registry Center (ChiCTR220058009, 26 March 2022). This study adhered to the STROBE guidelines. Written informed consent was obtained in accordance with the principles set forth in the Helsinki Declaration.

The study was conducted by recruiting patients at both centers between March 31, 2022, and June 30, 2022. The inclusion criteria were established as follows: participants had to be at least 18 years of age, undergo elective surgery, and receive intubation using a single-lumen endotracheal tube with VL under general anesthesia. Exclusion criteria included the cancellation of surgeries for various reasons, the presence of known difficult airways, psychiatric and neurological disorders, and refusal to participate by the patients.

General patient information was collected, including age, height, weight, body mass index (BMI), gender, and comorbidities such as diabetes, rheumatoid arthritis, cervical spondylosis, and neck scarring from various causes. Additionally, the American Society of Anesthesiologists (ASA) grades and surgery categories were recorded. Measurements of interincisor distance, thyromental distance, neck circumference, and Mallampati grades were performed by three trained independent investigators, who also evaluated whether neck movement was normal. Importantly, these three investigators were not involved in the follow-up process, ensuring that the anesthesiologists responsible for the perioperative management of the patients remained unaware of the specific details regarding these indicators. To minimize potential bias, Table 1 provides definitions for interincisor distance, thyromental distance, neck circumference, Mallampati grades, neck movement, and C-L classification [13]. In this study, difficult videolaryngoscopy is defined as a C-L classification of ≥ 3.

**Table 1:** Definition of measurement indices

Index	Definition
Interincisor distance, cm	The distance between the upper and lower central incisors at the maximum opening of the mouth (inter-gingival distance in edentulous patients)
Thyromental distance, cm	The distance from the notch of thyroid cartilage to the inner mentum when the neck is fully extended
Neck circumference, cm	Horizontal circumference of neck through thyroid cartilage
Mallampati grades (Open the mouth and extend the tongue as far as possible without pronouncing)	I: Soft palate, pharyngopalatine arch, uvula and hard palate visible
	II: Soft palate, uvula and hard palate visible
	III: Soft and hard palate visible
	IV: Only the hard palate visible
Neck movement	Flexion and extension 35 to 45 degrees, left and right rotation 60 to 80 degrees, left and right lateral bending about 45 degrees
C-L classification	I: The entire vocal cords are visible
	II: Parts of the vocal cords are visible
	III: The epiglottis is visible, but the vocal cords are not
	IV: Epiglottis is not even visible

*Abbreviations*: *C-L* Cormack-Lehane

All intubations were performed using a single model of hyperangulated videolaryngoscope (TD-C-IV, UE Medical Corporation, Zhejiang, China). The device was used with single-use, disposable, non-channeled hyperangulated blades (sizes 3 and 4 were available), which feature a 42-degree angulation at the distal tip. This design is comparable to other well-known hyperangulated VLs such as the GlideScope. The use of a stylet for tracheal tube shaping was a standardized and mandatory component of the intubation technique for all procedures, to ensure consistent and effective tube delivery.

All patients underwent routine preoperative visits conducted by a resident anesthesiologist. Anesthesia management was carried out collaboratively by an anesthesiology resident and an attending anesthesiologist. To minimize performance variability related to the learning curve, all resident anesthesiologists involved in intubations had completed a minimum of one year of clinical training that included the structured use of the specific VL model (TD-C-IV) employed in this study. The attending anesthesiologists supervising the procedures had at least three years of experience with VL. Upon entering the operating room, standard monitoring procedures were initiated for all patients, which include electrocardiogram, non-invasive blood pressure assessment, and pulse oximetry. Anesthesia induction was performed using a combination of sufentanil (0.4–0.5 µg.kg^− 1^), propofol (1.5–2.5 mg.kg^− 1^), and cis-atracurium (0.15–0.2 mg.kg^− 1^). Five minutes after the induction, the resident anesthesiologist employed VL to place a single-lumen endotracheal tube, which was pre-shaped with a stylet as per the standardized protocol. The patient’s head was positioned in the sniffing position. This position was standardized across the study as it is a familiar and routinely used posture for tracheal intubation in our clinical practice, which we found to be effective for obtaining a glottic view with the hyperangulated videolaryngoscope. A hyperangulated 3 blade was routinely utilized for intubation, with a hyperangulated 4 blade also available. For female patients, a 7.0 mm tracheal tube was preferred, while for male patients, a 7.5 mm tracheal tube was generally selected. Other models of endotracheal tubes were kept available as backups. In cases where patients present a C-L classification of ≥ 3 during intubation, several strategies were attempted sequentially: the BURP maneuver [14], the hyperangulated 4 blade, and a combination of the BURP maneuver with the hyperangulated 4 blade. Should intubation remain unsuccessful after these measures, alternative methods—including fiberoptic bronchoscopy guidance, laryngeal mask insertion, cricothyrotomy, or tracheostomy—were considered. The intubating anesthetist documented the patient’s C-L classification, noting whether the BURP maneuver and hyperangulated 4 blade were employed, the success of the VL intubation, and any remedial actions taken following unsuccessful VL intubation. Patients were categorized into two distinct groups based on their C-L classification: the easy videolaryngoscopy group (group E, classified as C-L I-II) and the difficult videolaryngoscopy group (group D, classified as C-L III-IV).

The SPSS 23.0 software was employed for the statistical analysis of the data. Measurement data exhibiting a normal distribution were reported as mean ± standard deviation. The comparison of means between the two groups was carried out using an independent samples *t*-test. For measurement data not conforming to a normal distribution, the median (interquartile range) was utilized, and group comparisons were conducted using the Mann-Whitney *U* test. Categorical variables were presented as counts (%), and the chi-squared test or Fisher’s exact test was applied for group comparisons. Potential factors associated with difficult video laryngoscopy were analyzed univariately, and those with a *P* value of less than 0.1 were subsequently included in a multivariate logistic regression analysis. Multicollinearity was assessed using the variance inflation factor (VIF) (VIF < 5 was considered no multicollinearity). A significance level of *P* < 0.05 was regarded as statistically significant.

## Results

**Fig. 1:**
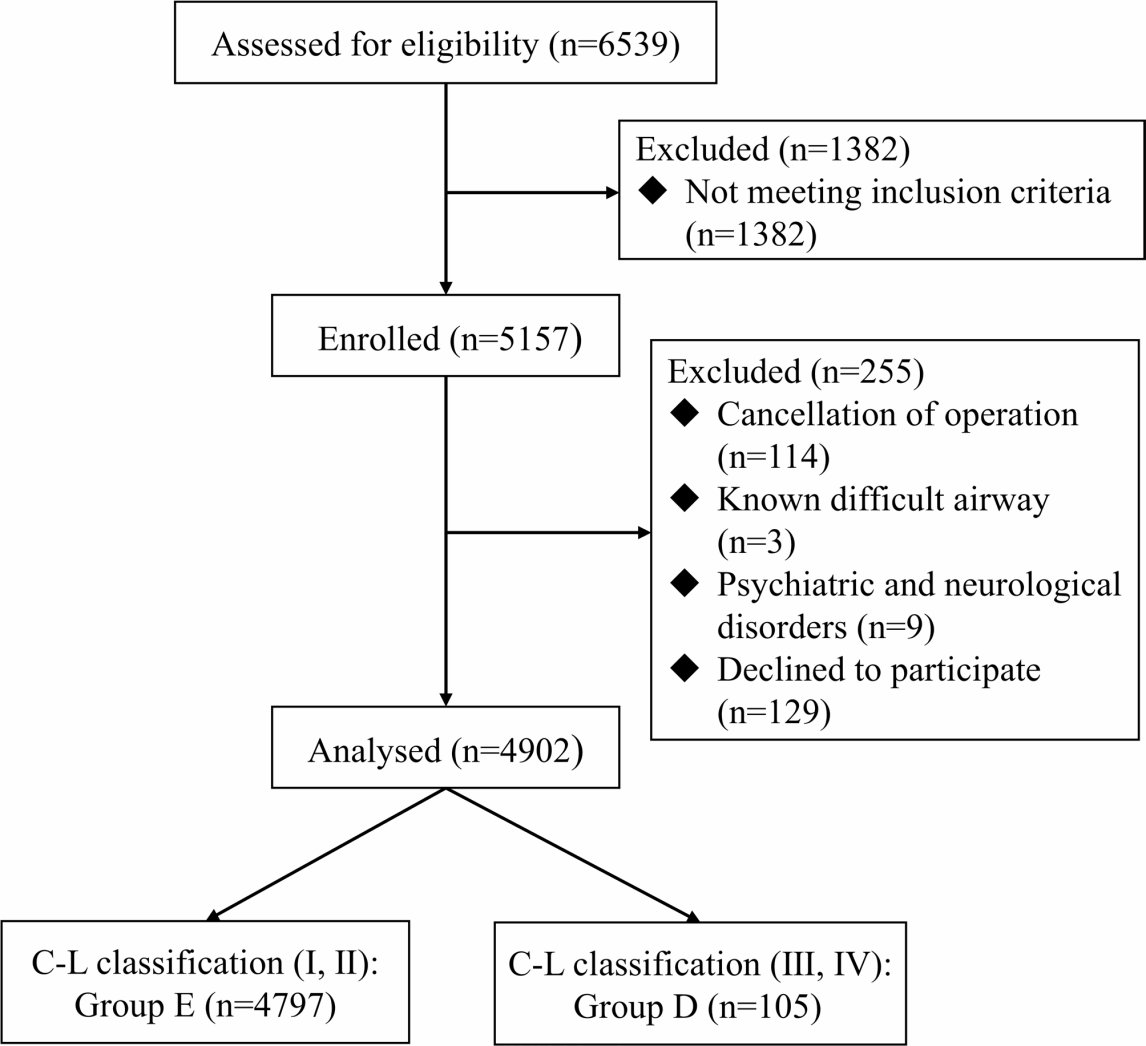
Flow diagram of patient recruitment

A total of 6,539 patients scheduled for elective surgery under general anesthesia were screened for inclusion in this study. Among these, 1,382 patients were excluded due to failure to meet the inclusion criteria, and 114 patients canceled their surgeries for various reasons. Additionally, three patients had a difficult airway, specifically two cases with vocal cord masses and one case of airway stenosis. Nine patients were excluded due to psychiatric and neurological disorders, while 129 patients declined to participate. Consequently, 4,902 patients were included in the final analysis, as illustrated in Fig. 1. The baseline characteristics of patients in both groups are presented in Table 2.

**Table 2:** Patient characteristics

Variables	E(*n* = 4797)	D(*n* = 105)	*P* value
Age, y	48 ± 14	52 ± 14	0.030
Height, cm	165 ± 8	167 ± 8	0.067
Weight, kg	69 ± 15	74 ± 17	0.002
BMI, kg.m^− 2^	25.2 ± 4.7	26.5 ± 5.1	0.006
Gender, n(%)			0.005
Male	2078 (43.3)	60 (57.1)	
Female	2719 (56.7)	45 (42.9)	
Comorbidities, *n*(%)			
Diabetes mellitus	439 (9.2)	9 (8.6)	0.838
Rheumatology or rheumatoid arthritis	45 (0.9)	1 (1.0)	>0.999
Cervical spondylopathy	323 (6.7)	4 (3.8)	0.235
Neck scar contracture	57 (1.2)	0 (0.0)	0.507
ASA grades, *n*(%)			0.006
I	1351 (28.2)	19 (18.1)	
II	2874 (59.9)	63 (60.0)	
III	512 (10.7)	21 (20.0)	
IV	60 (1.2)	2 (1.9)	
Surgery category, *n* (%)			
Thyroid surgery	1311 (27.3)	27 (25.7)	0.713
ENT surgery	1103 (23.0)	30 (28.6)	0.180
Gynaecologic surgery	407 (8.5)	5 (4.8)	0.174
Urology surgery	397 (8.3)	7 (6.7)	0.553
Gastrointestinal surgery	298 (6.2)	10 (9.5)	0.l67
Hepatobiliary surgery	278 (5.8)	9 (8.6)	0.231
Orthopedic surgery	205 (4.3)	4 (3.8)	>0.999
Bariatric surgery	166 (3.5)	5 (4.8)	0.653
Anorectal surgery	162 (3.3)	3 (2.8)	0.985
Neurosurgery	156 (3.3)	0 (0.0)	0.110
OM surgery	92 (1.9)	1 (0.9)	0.722
Breast surgery	75 (1.6)	0 (0.0)	0.374
Vascular Surgery	70 (1.5)	3 (2.9)	0.446
Ophthalmic surgery	40 (0.8)	1 (0.9)	0.590
Cardiac surgery	20 (0.4)	0 (0.0)	>0.999
Plastic surgery	17 (0.3)	0 (0.0)	>0.999
Interincisor distance, cm	4.3 ± 0.7	4.1 ± 0.7	0.001
Thyromental distance, cm	8.4 ± 1.2	8.3 ± 1.2	0.462
Neck circumference, cm	36.4 ± 4.0	37.8 ± 3.9	0.001
Mallampati grades, *n* (%)			<0.001
I	1822 (38.0)	18 (17.1)	
II	1115 (23.2)	12 (11.4)	
III	1080 (22.5)	45 (42.9)	
IV	780 (16.3)	30 (28.6)	
Neck movement, *n* (%)			>0.999
Normal	4744 (98.9)	104 (99.0)	
Limited	53 (1.1)	1 (1.0)	

Data shown by mean ± SD or numbers (%)

*Abbreviations*: *BMI* Body mass index, *ASA* American Society of Anesthesiologists, *ENT* Ear-nose-throat, *OM* Oral and maxillofacial, *E* Easy videolaryngoscopy, *D* Difficult videolaryngoscopy

Among the cohort of 4,902 patients, 4,797 (97.9%) were categorized as C-L grade I-II (group E), while 105 patients (2.1%) were classified as C-L grade III (group D). Notably, no C-L grade IV views (no glottic structures visible) were recorded in this study cohort. In group E, tracheal intubation was successful in all 4,797 patients. Among the 105 patients in group D, the sequential application of adjunctive maneuvers ultimately achieved successful intubation in 104 patients. Specifically, 81 were successfully intubated using the BURP maneuver in combination with a hyperangulated 3 blade, 17 were successfully intubated with a hyperangulated 4 blade, and six were successfully intubated by applying the BURP maneuver with a hyperangulated 4 blade. The sole failure in intubation was subsequently managed successfully with the use of a laryngeal mask.

Univariate analysis was conducted, as demonstrated in Table 2, identifying age, height, weight, BMI, gender, ASA grades, interincisor distance, neck circumference, and Mallampati grades as factors associated with difficult videolaryngoscopy (*P* < 0.1).

**Fig. 2:**
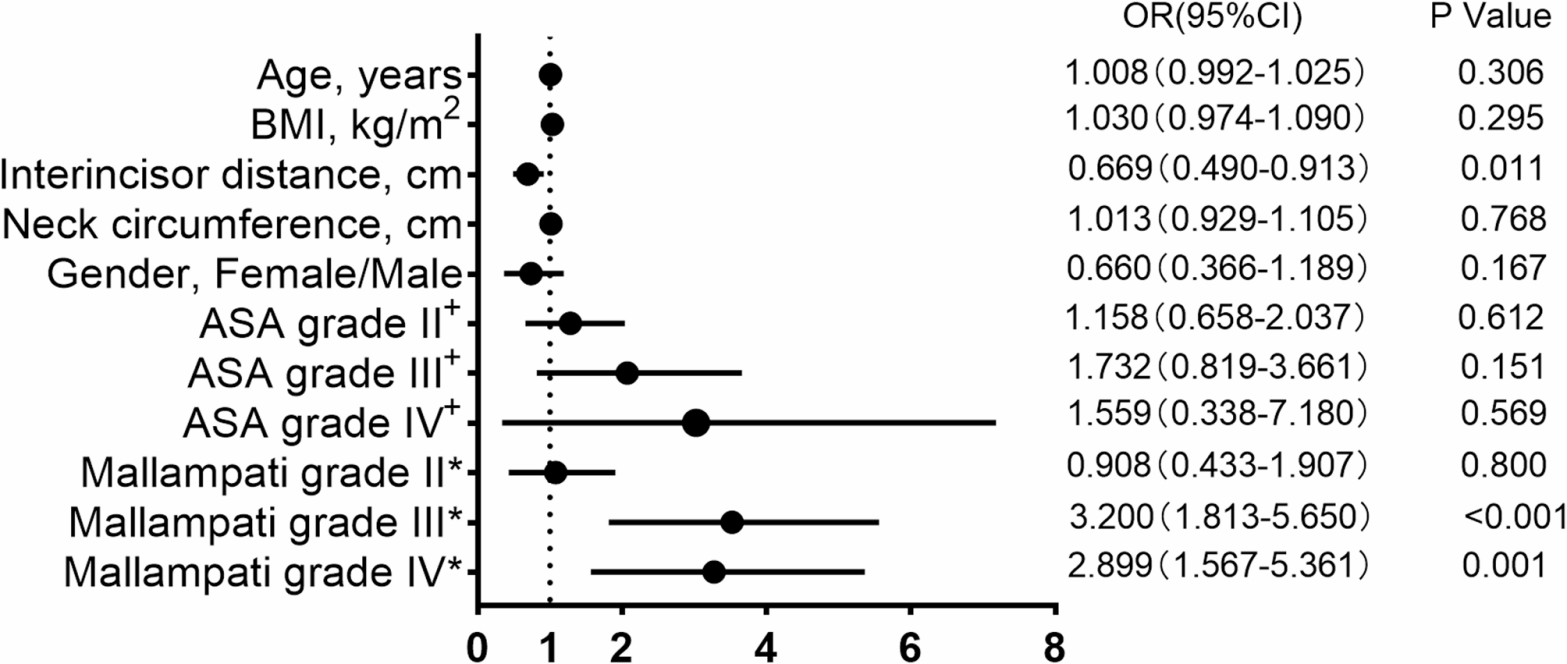
Forest plot of factors associated with difficult videolaryngoscopy. Odds ratio greater than 1 represented increased odds for difficult videolaryngoscopy. ^+^ Compared to ASA grade I, ^*^ Compared to Mallampati I. Abbreviation: BMI, body mass index; ASA, American Society of Anesthesiologists

A multifactor logistic regression analysis was conducted, which required the exclusion of height and weight due to multicollinearity, resulting in the exclusive retention of BMI for the multifactor assessment, as depicted in Fig. 2. The findings indicated that interincisor distance significantly influenced the occurrence of difficult videolaryngoscopy, with an OR of 0.669 and a 95% CI ranging from 0.490 to 0.913 (*P* = 0.011). Furthermore, there was a notable association between Mallampati grades III and IV and the likelihood of difficult videolaryngoscopy. Specifically, Mallampati grade III had an OR of 3.200 (95% CI:1.813–5.650, *P* < 0.001), while Mallampati grade IV showed an OR of 2.899 (95% CI:1.567–5.361, *P* = 0.001).

## Discussion

Our large-scale, prospective cohort study demonstrates that the incidence of difficult videolaryngoscopy with a hyperangulated device, defined as a C-L grade of III or higher, is 2.1% in a Chinese cohort of elective surgical patients with normal airway anatomy. Furthermore, we identified that a shorter interincisor distance and higher Mallampati grades (III and IV) are independent predictors for its occurrence. While these anatomical factors are known predictors for difficult direct laryngoscopy [15], our study provides robust, prospective confirmation that they remain relevant in the context of hyperangulated videolaryngoscopy. The primary contribution of this work is this large-scale, device-specific evidence. These findings are significant as they provide robust data on the challenges specific to videolaryngoscopy. The growing adoption of VL underscores the need to move beyond assumptions based on direct laryngoscopy and develop VL-specific evaluation criteria, as emphasized by recent calls for standardized videolaryngoscopy assessment frameworks [16]. The observed incidence of difficult videolaryngoscopy (2.1%) is substantially lower than the reported rates of difficult direct laryngoscopy, which can be as high as 9.7% in comparable populations [15]. This reduction is likely attributable to the superior glottic visualization afforded by the VL’s camera and screen, which bypasses many line-of-sight obstacles inherent to direct laryngoscopy. Importantly, nearly all cases of difficult videolaryngoscopy in our cohort were successfully managed with adjuvant maneuvers, such as the BURP technique or switching to a larger hyperangulated blade. This underscores that difficult visualization with a VL does not invariably lead to failed intubation, and highlights the importance of operator proficiency with these rescue strategies. The single intubation failure (0.02%) was successfully resolved with a supraglottic airway device, reinforcing the necessity of having a structured backup plan.

The very high ultimate success rate (99.98%) in our study should be interpreted in this context: it reflects a structured protocol in an elective surgical population, where failure was only declared after multiple adjunctive steps within the videolaryngoscopy system had been attempted. This differs from reports of first-attempt success rates in more heterogeneous or critically ill populations [17, 18], which are typically lower.

Among the anatomical predictors, interincisor distance was significantly associated with difficult videolaryngoscopy. A smaller interincisor distance limits the oral space available to maneuver the VL blade and achieve an optimal sightline to the glottis, even with an indirect video view. Our finding that an increased interincisor distance correlates with a reduced risk aligns with established literature on difficult direct laryngoscopy [15, 19–21], confirming that this fundamental anatomical constraint remains relevant in the videolaryngoscopy context. This warrant continued caution during the preoperative assessment of patients with reduced mouth opening.

Similarly, we found a strong, independent association between Mallampati grades III/IV and difficult videolaryngoscopy. This suggests that a crowded oropharyngeal anatomy, which obscures the view of the glottis during direct laryngoscopy, also presents a challenge for obtaining a clear view with a VL. While the predictive value of the Mallampati score has been debated [22, 23], our results indicate its sustained relevance. It is crucial that this assessment is performed correctly, with the patient seated and without phonation, to ensure grading accuracy and its utility in the VL era.

In contrast to the identified predictors, several factors were not independently associated with difficult videolaryngoscopy in our multivariate model. The ASA classification provides an overall health assessment and was not a specific predictor. While obesity is a known risk factor for difficult intubation with direct laryngoscopy [24], the average BMI in our cohort was not in the obese range, which may explain the lack of a significant association. Furthermore, neck circumference, which can correlate with difficult direct laryngoscopy [25], was not a significant predictor in our study. This discrepancy underscores a fundamental distinction between the two techniques. Recent research developing composite scores for difficult direct laryngoscopy has identified parameters like male gender and increased ultrasound-measured anterior neck soft tissue thickness as key predictors [26]. Videolaryngoscopy, by providing an indirect view of the glottis, often overcomes anatomical limitations imposed by anterior neck soft tissue that challenge direct line-of-sight techniques. Therefore, parameters strongly associated with difficult direct laryngoscopy are not necessarily transferable to the context of videolaryngoscopy.

This study has several limitations. Firstly, this study was conducted in a specific context: it investigated a single type of hyperangulated videolaryngoscope within a large academic population in China. As different VLs (e.g., those with Macintosh-style blades or channeled devices) have varying visualization angles and insertion mechanics, and as practice environments and patient demographics can differ significantly, our findings regarding the incidence and predictors of difficult videolaryngoscopy are most directly applicable to this specific device and setting. The results should not be overgeneralized to other VL designs, non-academic practice environments, or other ethnic populations. Secondly, our study relied on the C-L classification to define the primary outcome. While it is a well-established system, it was designed for direct laryngoscopy and has conceptual limitations when applied to videolaryngoscopy. The C-L grade focuses solely on the degree of glottic visualization and does not account for potential difficulties in tracheal tube delivery, which is a recognized point of failure in VL even with an adequate view [27]. Future research should aim to adopt grading standards specifically designed for videolaryngoscopy. For instance, the recently proposed Video Classification of Intubation (VCI) score provides a multidimensional framework that integrates blade type, the Percentage of Glottic Opening (POGO) score, and tube delivery outcome, potentially offering a more comprehensive assessment for VL studies [28]. Thirdly, our study focused on difficult glottic exposure (C-L grade ≥ 3) and did not systematically analyze the “can see but can’t intubate” scenario, a recognized phenomenon in videolaryngoscopy where excellent glottic visualization does not guarantee easy tracheal tube passage [27]. We did not record data such as the POGO score or specific difficulties in tube delivery despite a good view, which precluded a sub-analysis of this important clinical challenge. Fourthly, although all operators met a minimum experience threshold with the device, a range of proficiency existed, and we did not analyze its impact as a separate variable. This could be a source of variability in glottic visualization and is an important factor for future research to consider. Fifthly, while our study focused on difficult glottic exposure, it is important to contextualize its predictive value for the ultimate endpoint of failed intubation. It is well-established that a poorer glottic view (e.g., C-L grade ≥ 3) is associated with a lower first-pass success rate [3, 29]. However, in our cohort, which employed a structured protocol with sequential adjunctive maneuvers (BURP, blade change), a poor glottic view was a poor predictor of ultimate intubation failure, as nearly all such cases (104/105) were successfully managed within the videolaryngoscopy strategy. Therefore, while a difficult VL view reliably predicts a more challenging intubation process, its predictive value for the outcome of absolute failure may be limited in settings where systematic rescue techniques are immediately available and effectively applied. Sixthly, although we recorded the presence of comorbidities and assessed neck mobility, our analysis did not account for the severity of these conditions, which might offer further predictive insights. Finally, our study focused on the outcome of glottic view and ultimate intubation success. Consequently, we did not collect several established intubation process metrics, such as first-pass success rate, the number of intubation attempts, time to intubation, or specific complications like hypoxemia, esophageal intubation, or airway trauma. The absence of these data limits a more granular analysis of the intubation process and precludes comparison with studies that use these metrics as primary endpoints. Future research on videolaryngoscopy would benefit from the systematic inclusion of these quality indicators to provide a more comprehensive assessment of performance and safety.

## Conclusions

The incidence of difficult videolaryngoscopy was 2.1% in this cohort when using a hyperangulated videolaryngoscope. The interincisor distance and Mallampati grades III and IV are independently associated with the occurrence of difficult videolaryngoscopy with this blade type.

## Data Availability

Datasets can be obtained from the corresponding author upon reasonable request, subject to ethical and legal restrictions.

## Abbreviations

VL: Video laryngoscope
CL: Cormack-Lehane
BMI: Body mass index
ASA: American Society of Anesthesiologists
VIF: Variance inflation factor
